# The link between varicella and immune system: which children will develop acute cerebellitis?

**DOI:** 10.1186/s13052-020-00840-5

**Published:** 2020-05-29

**Authors:** Elena Bozzola, Rita Carsetti, Eva Piano Mortari, Marco Masci, Giulia Spina, Alberto Villani

**Affiliations:** 1 Pediatric and Infectious Diseases Unit, University/hospital Department of Pediatrics, Bambino Gesù Children’s Hospital, IRCCS, Rome, Italy; 2 B cell Physiopathology Unit, Immunology Research Area, Bambino Gesù Children Hospital, Rome, Italy

**Keywords:** Varicella, Children, Cerebellitis, Immune system, Vaccine

## Abstract

**Introduction:**

Varicella may complicate with cerebellitis in previously healthy children, requiring hospitalization. Aim of our study was to define whether children who experienced varicella cerebellitis have a normal immune system.

**Methods:**

Patients over 3 years of age admitted at Bambino Gesù Children from January 2006 till June 2016 for cerebellitis in varicella were asked to participate to the follow-up study. The immune status was evaluated clinically and by laboratory investigations.

**Results:**

Twenty-five patients were included in the study. At follow up, at least one immunological alteration was detected in 80% of patients. To avoid bias due to possible effects of the recent disease, we separately analyzed patients who had the follow-up control at least 1 year (Group 1) or between 1 month and 1 year (Group 2) after the hospitalization for acute varicella cerebellitis. The results were similar in both groups with immunological alterations detected in 84,6 and 75% of the patients, respectively.

**Conclusions:**

Our preliminary results indicate that sub-clinical immunological defects may correlate to cerebellitis in varicella.

## Background

Varicella (VZV) is an exanthematous infectious disease that mainly occurs in the pediatric age. Despite the public perception of varicella infection as a harmless childhood affliction, the clinical course can be severe [1]. In fact, Chickenpox may require hospitalization and relevant medical care services [2].

In 2017, the varicella vaccine was introduced as a mandatory vaccination in Italian children. A universe immunizationis expected a reduction of the incidence of varicella and cases requiring hospitalizations, as well as in other countries [3].

Varicella-related hospitalization rates differ widely worldwide. Varicella potentially afflicts every organ: haematological, neurological, respiratory, cutaneous, hepatic, gastrointestinal, urinary, and bone complications are the most frequently reported [4]. The incidence of varicella complications differs among scientific reports. The pooled prevalence of neurological complications resulting from a systematic review of the literature identifies the likelihood of pediatric neurological complications in the range of 13.9–20.4% [5]. In children affected by varicella, prompt antiviral therapy may be indicated to reduce the number of days of hospitalization [6].

Nevertheless, in literature a considerable rate of neurological sequelae (5%) in acute cerebellitis and in acute cerebellar ataxia had been reported, even after antiviral and steroid therapy [7]. Neurological complications of VZV infections can be categorised into those caused by the primary infection and those associated with an immunomediated inflammation triggered by the infection [8].

The pathogenesis of neurological complications in varicella is unknown. Viral invasion of the central nervous system or autoimmune processes have been hypothesized [9].

The immune system plays an important role in the defense against varicella. Patients with a history of underlying malignancy, steroid use, immunosuppressive therapy, HIV infection, or solid organ transplantation are susceptible to disseminated varicella and to varicella reactivation with or without neurological complications [10]. Moreover, varicella vaccines inducing strong and persistent B and T cell immunity effectively protect children and adults from infection [11].

### Aim of the study

Aim of our study is to define if children who experienced a varicella cerebellitis have a normal immune system.

## Material and methods

For the purpose of the study, we enrolled patients admitted at Bambino Gesù Children Hospital for cerebellitis in varicella from January 2006 till June 2016.

In our previous study, we defined the characteristic of acute cerebellitis in varicella among hospitalized children [12].

Criteria for inclusion were age at diagnosis over 3 and under 18 years. At least 1 month after hospital discharge for varicella, families were contacted by phone and invited to our outpatient clinic for a 1 day evaluation. The following procedures were performed: pediatric infectious visits, vaccine check and laboratory exams to investigate the immune status.

In detail, laboratory tests included: measurement of antibodies against vaccine antigens (tetanus, *Haemophilus influenzae B*, *Streptococcus pneumoniae*, *Bordetella pertussis* and Hepatitis B), serum immunoglobulin concentration (IgM, IgA, IgG), evaluation of lymphocyte subpopulations (CD3, CD4, CD8, central and effector memory T cells, CD16/56, CD19, transitional, mature naïve and memory B cells) and in vitro antibody production.

We excluded from the study children who at diagnosis: 1) were under 3 years of age, 2) were affected by immunodeficiency, chronic diseases or malignancy and 3) had received immunosuppressive therapy before the blood sample.

An informed consent was obtained by the parents.

## Results

Twenty-five patients were included in the study. At the time of acute hospitalization for varicella, patients were not vaccinated for VZV. No gender difference was observed (48% female, 52% male). The mean age at hospital admission for acute cerebellitis in varicella was 5.79 years (SD 0.33) and the mean age at the outpatient visit was of 7.82 years (SD 2.01) (Table 1).

At the outpatient control, all patients were in good clinical conditions, without either fever or infectious disease. The medical history was collected for each patient and was negative for recurrent or severe infection, except for varicella cerebellitis. Certificates of vaccination were regular, according to chronological age.

Nevertheless, immunological laboratory exams were altered in most of the patients. In order to avoid bias due to possible effects of the recent disease, we separately analyzed patients who underwent the outpatient control at least 1 year (Group 1) or between 1 month and 1 year (Group 2) after the hospitalization for acute varicella cerebellitis. Sub-lymphocyte and immunoglobulin values were studied considering the two groups (Table 2).

**Table 1:** Clinical and laboratoristic data

Parameters	
Sex (F/M) %	48%/52%
Mean age at hospital admission	5.79 years
Mean age at follow up (years)	7.82 years
CRP	< 0.5 mg/dl
ESR	20 mm/h

CRP (normal value < 0.5 mg/dl); ESR (normal value < 20 mm/h)

**Table 2:** Sub-lymphocyte and Ig values in group 1 and 2

**Group1**	**CD3**	**CD4**	**CD8**	**CD19**	**CD16/56**	**IgA**	**IgG**	**IgM**
**mean**	67,489	35,322	24,411	19,478	12,8	137,1	974,2	186,5
**SD**	47,996	4904	38,566	44,726	4,569,737	66,786	368,94	225,52
**min**	59,8	25	16,4	11,2	7,3	19	252	46
**max**	73,4	40,2	29,3	27,5	20,5	207	1420	815
**Group2**	**CD3**	**CD4**	**CD8**	**CD19**	**CD16/56**	**IgA**	**IgG**	**IgM**
**mean**	61,253	32,284	21,579	17,886	12,11,543	120,24	871,43	237,59
**SD**	19,345	10,603	71,464	68,703	5,623,631	70,987	423,98	276,06
**min**	58,2	30,9	17	8,9	5	19	670	49
**max**	84,5	49,3	38,3	26,5	23,7	138	1445	246

Group 1 included 13 patients. Eleven of them (84.6%) had at least one immunological alteration. Nine of the 12 children of Group 2 (75%) had altered immunological parameters.

In details, in Group 1, 10 out of 13 patients did not reach protective level of specific antibodies for at least one of the evaluated antigens against which they had been vaccinated before. Three of 13 children had reduced or absent in vitro antibody production and 2 of 13 had a decreased number of switched memory B cells. Five children had multiple defects.

Similar immunological impairments were detected in Group 2. Insufficient response to vaccination was observed in eight patients, low/absent in vitro antibody production was observed in five and switched memory B cells were reduced in three patients. Finally, multiple defects were observed in five children.

**Fig. 1:**
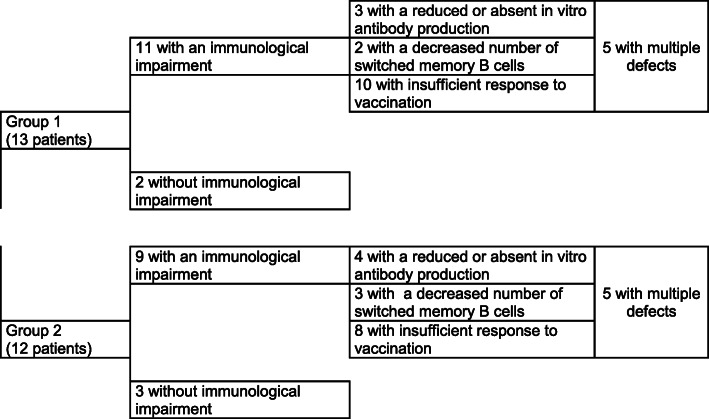
Immunological results in patients hospitalized for varicella cerebellitis

Figure 1 summarizes the results.

## Discussion

Most of the patients (80%) previously affected by cerebellitis in varicella presented with at least an immunological alteration detected by laboratory tests. As the tests were performed at least 1 year after diagnosis in children of Group 1, the immunological alterations were considered persistent. None of the children had clinical signs of immune deficiency indicating that the variation in their immune function may influence the severity of varicella rather than the susceptibility to infection. Varicella has usually a benign course in immunocompetent children. On the contrary, individuals with immune responses significantly below “normal” are more susceptible to infectious agents and exhibit increased infectious morbidity and mortality. Our results are similar to literature, considering VZV-infections in immunocompromised patients a serious health problem related to morbidity and even death [13].

In particular, a previous study focused on a 6-year analysis of hospitalization and complication rates of VZV-infection in immunocompromised patients [14]. The researchers conclude that early antiviral treatment and varicella-zoster immunoglobulin were successful in reducing complications and mortality rate (< 1%) in the affected patients with immune suppression compared to healthy group [14].

Furthermore, routine vaccination against varicella has the potential role to diminish the number of infections and complications but also to produce herd immunity, essential for immunocompromised children [15].

## Conclusion

Our preliminary results may indicate that subclinical, but measurable, immunological alterations may correlate to cerebellitis in varicella. Otherwise, immunocompromised children who experience varicella have higher risk to undergo complications and a severe course of the disease. For this reason, a prompt therapy should be performed.

Our study has some limits. One limit is that there is not a control group represented by children who developed a mild form of varicella as we cannot consider ethical to prescribe blood controls without a suspicion of defect. Normality values for immunocompetent children of comparable age were obtained from the literature [16, 17]. Another potential limit is the small sample size due to the rarity of cerebellitis in varicella among immunocompetent children. It is, however, important to underline the high frequency of persistent immunological alterations in the analyzed patients. Further study are need to enforce our conclusions.

Finally, VZV has developed multiple mechanisms to block the induction and perpetuation of both innate and adaptive immune responses even if it presents with a mild clinic form. Further studies may be useful to examine the effects of mild varicella infection to the immune system during and after active infection.

## Data Availability

At Bambino Gesù Children Hospital

## Abbreviation

VZV: Varicella
